# Patterns of Children’s Relationships With Parents and Teachers in Grade 1: Links to Task Persistence and Performance

**DOI:** 10.3389/fpsyg.2022.836472

**Published:** 2022-05-19

**Authors:** Gintautas Silinskas, Eve Kikas

**Affiliations:** ^1^Department of Psychology, University of Jyväskylä, Jyväskylä, Finland; ^2^School of Natural Sciences and Health, Tallinn University, Tallinn, Estonia

**Keywords:** parent–child relationship, teacher–student relationship, task persistence, performance, Grade 1

## Abstract

Our study aimed to investigate the patterns of children’s relationships with their parents and teachers, the development of these relationships during Grade 1, and respective links to children’s learning (in task persistence and performance). Parents of 350 children answered questionnaires about the quality of their relationships with their children; 25 teachers answered questions about children’s task persistence at school and the quality of their relationships with their students; 350 children completed literacy and math performance tests; and six testers evaluated children’s task persistence when completing those tests. All measures were administered twice: at the start and end of Grade 1. Latent profile analyses found two meaningful child profiles that were similar at the beginning and end of Grade 1: *average relationship* (89% at T1, 85% at T2) and *conflictual relationship* (11% at T1, 15% at T2) with parents and teachers. These profiles were highly stable throughout Grade 1, except for 15 children who moved from an *average relationship* to a *conflictual relationship* profile. This *declining trajectory* can be characterized by poor relationships with teachers and low task persistence at the end of Grade 1, although they did not perform any worse than other children. Finally, children exhibiting conflictual relationships with their parents and teachers at the beginning of Grade 1 performed worse on spelling and subtraction tasks and demonstrated lower task-persistent behavior at the end of Grade 1 than those with average (good) relationships with parents and teachers.

## Introduction

Children’s development takes place in close interaction with home and school environments (Bronfenbrenner, 1979; Bronfenbrenner and Morris, 2006). The role of parents and teachers in children’s learning becomes critical upon entering school (Heatly and Votruba-Drzal, 2017), when children continue to be educated by their primary socializers (parents) and new relationships with teachers are formed. Relationships with parents and teachers in tandem contribute to children’s sense of belonging (Ryan and Deci, 2000, 2020), engagement with learning (e.g., task persistence), and academic skills (e.g., reading, spelling, and math). Moreover, children’s personal characteristics (e.g., task persistence and skills) evoke certain instructional and emotional reactions from their socializers (Silinskas et al., 2013, 2015, 2016) and establish relationships of a certain quality (Heatly and Votruba-Drzal, 2017). Relationships formed in Grade 1 provide the foundation upon which children build later relationships (Heatly and Votruba-Drzal, 2019). Thus, understanding the interplay between the quality of children’s relationships with their parents and teachers and how it relates to learning at the very start of their school career (Grade 1) is crucial to preventing challenges in school adjustment beyond Grade 1.

Existing research on this topic has several limitations. First, previous studies have mostly investigated the associations between the relationship quality of only one interpersonal environment (parents or teachers) and children’s learning outcomes, whereas children’s development takes place in interaction with both parents and teachers (for exceptions, see Pianta et al., 1997; Furrer and Skinner, 2003; Heatly and Votruba-Drzal, 2017, 2019). Second, the majority of studies have investigated either adolescents’ relationship quality (Seiffge-Krenke et al., 2010) or children too young for school (Cook et al., 2012). However, relationship patterns at the transition to primary school and during Grade 1 are of particular importance because these set the stage for further development of relationships and learning outcomes (Heatly and Votruba-Drzal, 2017). Third, little is known about whether child characteristics like task persistence and performance act as predictors or outcomes of patterns related to the quality of children’s relationships with their parents and teachers. Longitudinal studies are necessary to answer these questions. Fourth, to the best of our knowledge, person-oriented approaches have not been used to examine the possibility of different relationship patterns and their associations with child characteristics and development (Bergman et al., 2003). Person-oriented analyses might clarify earlier mixed findings on the joint effect of parent and teacher relationships on children’s development (e.g., Heatly and Votruba-Drzal, 2017, 2019). Finally, as parent–child and teacher–student relationships are embedded in/affected by wider cultural and educational contexts (Bronfenbrenner, 1979; Bronfenbrenner and Morris, 2006), evidence from different countries could enhance the generalizability of findings across cultural environments. Accordingly, the present study was set up to investigate profiles and trajectories of Lithuanian children’s relationships with parents and teachers over the course of Grade 1 and how these relate to their task persistence and performance.

### Children’s Relationships With Parents and Teachers

In the present study, the quality of relationships between children and their significant adults (interpersonal environment: parents and Grade 1 teachers) is understood in terms of closeness and conflict. *Closeness* may be defined as emotionally positive and open communication and a sense of shared affection and warmth (Pianta, 1999; Hamre and Pianta, 2001; Pakarinen et al., 2017; Heatly and Votruba-Drzal, 2019; Valiente et al., 2019). In contrast, *conflict* can be defined as the manifestation of negativity, argumentative exchanges, hostility, and struggle managing children’s behavior positively or proactively (Pianta, 1999; Heatly and Votruba-Drzal, 2019). Longitudinal studies show that close and conflictual relationships with parents and teachers tend to be relatively stable through the primary school years (Skinner and Belmont, 1993; Hughes, 2011; Heatly and Votruba-Drzal, 2017). Moreover, children’s relationships with their parents can affect that with their teachers. For instance, children in conflictual relationships with parents in preschool tend to develop conflictual relationships with their Grade 1 teachers (Heatly and Votruba-Drzal, 2017), and children with good relationships with their parents in preschool tend to have close relationships with their Grade 1 teachers (Pianta et al., 1997; Pianta and Stuhlman, 2004; Heatly and Votruba-Drzal, 2017).

So far, investigations into the development of children’s relationships with parents and teachers simultaneously have been rare (for an exception, see Heatly and Votruba-Drzal, 2019), and none has applied a person-oriented approach to provide information on groups of children rather than associations between variables. Person-oriented studies investigating parents usually focus on parent–child relationships of adolescents (Seiffge-Krenke et al., 2010) or young children (Cook et al., 2012). Studies that include first-graders in their examination of the development of trajectories of the teacher–student relationship are more common than for the parent–child relationship. For instance, Miller-Lewis et al. (2014) followed teacher reports of their relationships with children throughout preschool to Grade 2 and found two trajectories: stable-high quality and moderate declining quality of overall relationship scores (in terms of closeness and reversed conflict). Other studies typically find more than two trajectories (O’Connor et al., 2011; Spilt et al., 2012; Valiente et al., 2019). Taken together, relationship-trajectory research emphasizes the stability of such trajectories but reports mixed results concerning their number. These mixed results partly depend, for example, on the length of the period under investigation and whether the study includes a composite score for relationship quality (versus investigating dimensions separately). However, neither approach has thus far included children’s relationships with both their parents and teachers. Thus, our longitudinal study of Grade 1 students investigated separate dimensions of children’s relationships (i.e., closeness and conflict) with their parents and teachers over the course of Grade 1 using a person-oriented method.

### Relationship Quality With Parents and Teachers and Children’s Task Persistence

A key aspect of children’s learning is their motivation and engagement with the learning process. In this article, we examined students’ task persistence, which is an indicator of both motivation and engagement (cf. Mägi et al., 2018). Task persistence has been defined as perseverance with school tasks or making an effort to complete even the most difficult tasks without giving up (Onatsu-Arvilommi and Nurmi, 2000; Kikas et al., 2014). It is related to effortful control of behavior, activating behaviors aimed at task completion, and inhibiting impulses unrelated to task behaviors (e.g., Mägi et al., 2018). Students who exhibit high task persistence carry on with a task even if it is challenging, while students with low task persistence (also interpreted as task avoidance) give up on a difficult task, try to find less-challenging tasks, or turn their attention to something more interesting (Kikas et al., 2014).

A plethora of studies over the decades show that parent–child relationship quality predicts a child’s engagement and persistence (e.g., Estrada et al., 1987; Pianta et al., 1991; Gregory and Rimm-Kaufman, 2008). Other studies have highlighted the importance of emotionally supportive and non-conflictual teacher–child relationships to a child’s development (for an overview from preschool to high school, see Roorda et al., 2011; for the mathematics context, see Yang et al., 2021). Significant associations between task persistence and teacher–student relationships have also been found among kindergarten and preschool-aged children (Lippard et al., 2017; Papadopoulou and Gregoriadis, 2017) and at the beginning of schooling. For instance, in one study, teachers reported higher task persistence for Grade 1 and Grade 3 students who perceived higher support and affection from teachers (Kikas and Tang, 2019).

Fewer studies have examined the effects of parent–child and teacher–child relationships simultaneously, yielding mixed findings. In preschool children, Pianta et al. (1997) found that both parent–child and teacher–child relationships—being moderately associated—were related to kindergarten adjustment. However, teacher–student relationships became unrelated to engagement after accounting for the parent–child relationship at school entry. In contrast, Furrer and Skinner (2003, a study of primary school students) found that children’s relatedness with parents and teachers, respectively, was positively related to engagement. Heatly and Votruba-Drzal (2017, 2019) examined, in two longitudinal studies, the integrative effects of parent–child and teacher–child relationships. In the first study, parent–child relationships were assessed at two time points (54 months and the beginning of school) and teacher–child relationships at the beginning of school. They found that maternal closeness and conflict predicted closeness and conflict with teachers and that teacher conflict, in turn, had a direct negative effect on engagement. The parent–child relationship was not found to be related to engagement at the beginning of school. However, it was found that parental closeness is important for engagement at school entry when children experience conflict with their teachers. Thus, they argued that parental closeness might protect children against negative relationships with teachers. In their second study, Heatly and Votruba-Drzal (2019) examined how children’s relationships with parents and teachers in Grades 1, 3, and 5 are associated with engagement and motivation in Grade 5. They found that only conflictual relationships with teachers predicted lower engagement. Thus, the integrated roles of parent–child and teacher–child relationships may be complex, and their combined effects on child outcomes may depend on a child’s age, the method of study, as well as the interaction of the two relationships. None of the reviewed studies applied a person-oriented approach to address this topic; thus, it was selected as the aim of our study.

### Relationship Quality With Parents and Teachers and Children’s Academic Performance

Another important factor in children’s learning is their academic performance. Reading, spelling, and math (i.e., addition and subtraction) skills are essential academic skills from the start of Grade 1 onward. Early reading and spelling skills become relatively stable at the beginning of primary school and continue to improve thereafter (Entwisle et al., 2005; Duncan et al., 2007); the same is true for math skills (Aunola et al., 2004). Conceptually, it has been suggested that in addition to general instructional support, emotional support is also important to meet students’ needs for belonging and competence (Ryan and Deci, 2000, 2020), which, in turn, enhances academic skills (see Hamre and Pianta, 2001).

Several studies have shown a relationship between the quality of children’s relationships with parents at home and teachers at school and the development of academic performance (Silinskas, 2012, Silinskas et al., 2017; Nurmi et al., 2018). In particular, research into children’s relationships with teachers typically reports positive associations between close/non-conflictual teacher–student relationships and students’ academic performance in Grade 1 and beyond (Hughes, 2011; Roorda et al., 2011; Lippard et al., 2017; Valiente et al., 2019). For instance, teachers tend to rate their Grade 1 students’ academic performance higher if they feel that the teacher–student relationship is close but lower if it is conflictual (Pianta and Stuhlman, 2004). Other studies, however, have not found any association between the teacher–student relationship and academic performance (Maldonado-Carreno and Votruba-Drzal, 2011; Hajovsky et al., 2017). Some findings are domain specific. For instance, McCormick et al. (2013) found relationship quality in kindergarten to relate to math but not reading performance, whereas Ly et al. (2012) found that closeness/conflict was related to reading performance (Ly et al., 2012).

A person-oriented approach was used to explore different profiles of parenting practices (including warmth, affection, and negative regard) and their relationship with child cognitive outcomes at age 3 (Cook et al., 2012). However, no studies have investigated the trajectories of parent–child relationship quality in relation to performance in early primary school. In contrast, teacher–student relationship trajectories have been studied in relation to reading and math performance from kindergarten to Grade 2 (Valiente et al., 2019) and to Grade 6 (Bosman et al., 2018). In particular, Valiente et al. (2019) followed students from kindergarten through Grades 1 and 2 and found a tendency toward higher performance in reading and math in children on increasing closeness/decreasing conflict trajectories compared to those on decreasing closeness/increasing conflict trajectories. So far, no studies have examined profiles of teacher–child and parent–child relationships (in terms of closeness and conflict) in relation to children’s performance from the beginning to end of Grade 1. Accordingly, we adopted this as one of the aims of our study.

### Evocative Effect of Children’s Task Persistence and Performance on Their Relationships With Parents and Teachers

Although relationships with parents and teachers are important in promoting children’s learning (including task-persistent behavior and academic performance), the opposite can also be true. These ideas are postulated by transactional theories of child socialization (Sameroff, 2010) and studies on the evocative effect of children’s characteristics on adults’ behaviors (Scarr and McCartney, 1983; Nurmi, 2012). Parents and teachers may respond differently to children who enter Grade 1 with low engagement and poor academic skills (Hoover-Dempsey and Sandler, 1995; Sameroff, 2010).

At home, parents of low-performing and task-avoidant children tend to respond with more controlling practices and higher negative affect in learning/homework situations (Pomerantz and Eaton, 2001; Silinskas et al., 2015), which can result in harsher relationships with children. Indeed, previous research shows that cognitive skills in preschool (measured as a composite score including letter identification, numbers/counting, comparison, etc.) can predict maternal closeness and sensitivity (Heatly and Votruba-Drzal, 2019). At school, children’s early task persistence and performance has been associated with the ability to initiate and sustain engagement with academic materials and, thus, related to better relationships with the teachers at school entry (Heatly and Votruba-Drzal, 2017, 2019). Studies have shown that high-performing students receive more emotional support and positive affect from their teachers than their lower-performing peers (Babad, 1990; Nurmi, 2012). For instance, Heatly and Votruba-Drzal (2019) found that cognitive skills in preschool predicted teacher closeness in Grade 1. Moreover, some studies differentiate between various subject matter. For instance, reading comprehension in Grade 3 predicted student-perceived closeness and teacher-perceived conflict in Grade 6 (Zee et al., 2021), whereas, in the other studies, math performance (not reading) predicted teacher–student closeness and conflict (Hajovsky et al., 2017). Even though these relationships have been demonstrated in variable-oriented approaches, similar trends and domain specificity remain to be investigated *via* a person-oriented approach.

### Lithuanian Educational System

Lithuanian children enter Grade 1 on the first of September of the calendar year of their seventh birthday, after a full year of kindergarten (LR Ministry of Education and Science, 2014). The aim of kindergarten education in Lithuania is to ensure the optimal development of the child’s individual qualities and to prepare him or her to learn according to the primary-education curriculum (LR Ministry of Education and Science, 2014). However, the kindergarten curriculum has no established criteria for determining the levels of a child’s reading, spelling, or math skills before school entrance. It is only in Grade 1 that teachers expose their students to the systematic teaching/learning of reading, spelling, and math. In addition, when children enter Grade 1, they often change school buildings and almost always get a new teacher, who is assigned to the class for the duration of primary school (Grades 1–4). This can also entail a new composition of the classroom, as new classes are formed during this transition. Accordingly, our study captured this critical and challenging transition in childhood when new relationships with teachers are formed and the systematic teaching of reading, spelling, and math begins.

### Research Questions

The main aim of the current study was to examine profiles of first-graders’ relationships with their parents and teachers, the continuity or change in these profiles over the Grade 1 school year, and how children’s learning (in terms of task persistence and performance) relates to these profiles and their trajectories. We utilized a person-oriented analytical approach and a longitudinal design that employed reports by parents, teachers, children, and testers. The main research questions (RQs) and hypotheses are as follows.

First (RQ1), what latent profiles based on parent- and teacher-reported relationship quality (in terms of closeness and conflict) exist, and how stable are they during Grade 1? What changes occur between the beginning of Grade 1 (T1) and the end of Grade 1 (T2)? As there is little previous research in this area, and since what there is reports mixed findings for the teacher–student relationship (including different numbers of profiles and their transitions, none of which include parents), we based our expectations on variable-oriented research and person-oriented research of children’s relationships with teachers. We expected that the largest profile would be adaptive and consist of students who enjoy a good (average) quality of relationships with both their parents and teachers (Spilt et al., 2012; Miller-Lewis et al., 2014; Heatly and Votruba-Drzal, 2017; Valiente et al., 2019). Additionally, we expected to find one or more non-adaptive profiles (Spilt et al., 2012; Miller-Lewis et al., 2014; Heatly and Votruba-Drzal, 2017; Valiente et al., 2019). For example, we expected to find one profile of child–parent relationships exhibiting lower-than-average closeness and higher-than-average conflict; one profile of child–teacher relationships exhibiting lower-than-average closeness and higher-than-average conflict; and one profile exhibiting lower-than-average closeness and higher-than-average conflict in both types of relationship. We further expected that children would mainly stay in similar profiles from the beginning to the end of Grade 1 but that several would move from an adaptive to a non-adaptive profile (i.e., non-adaptive transitions) while others moved from a non-adaptive to an adaptive profile (i.e., adaptive transitions) over the course of Grade 1.

Second (RQ2), to what extent do children’s transitions during the Grade 1 school year differ in terms of child task persistence and performance? We expected that task persistence and performance would be higher in any adaptive transitions versus non-adaptive transitions.

Third (RQ3), to what extent can children’s task persistence and performance at the end of Grade 1 be predicted by the profiles of children’s relationship quality at the start of Grade 1 (T1)? We expected that children reflecting the adaptive profile at the start of Grade 1 would exhibit higher task persistence and better performance (i.e., in reading, spelling, addition, and subtraction) at the end of Grade 1.

Earlier studies have demonstrated differences in the quality of children’s relationships with parents and teachers based on parental educational level (Roorda et al., 2011; Zee et al., 2021) and child gender (Roorda et al., 2011; Valiente et al., 2019). Thus, we explored whether transitions would differ in relation to parental educational level and child gender, and we controlled for parent education and child gender in predicting child outcomes.

## Materials and Methods

### Participants and Procedures

The data for the current study came from the longitudinal data collection (Silinskas and Raiziene, 2017–2018). Data on Lithuanian children, their parents, and their teachers were collected twice: once at the beginning of Grade 1 (T1) and again at the end of Grade 1 (T2). Participants in the current study came from six schools representing urban (65%) and rural (35%) distributions of the Lithuanian population. The principals of all schools contacted agreed that data collection could take place on their school premises. Grade 1 teachers were asked to complete questionnaires and help with distributing questionnaires to their students’ parents. School psychologists were trained to administer the tests and tested each child individually. All participating parents were introduced to the study’s goals and signed informed consent forms for their own and their children’s participation.

All main variables were collected twice—once at the beginning of Grade 1 (T1) and again at the end of Grade 1 (T2). A total of 350 children (53.4% girls and 46.6% boys), their parents, 25 teachers, and six testers (school psychologists) took part in our longitudinal investigation. The mean age of the children at T1 was 7.3 years (SD = 0.38). The majority of the children lived with both of their parents (79.6%); 11.0% with their mother only; 4.5% with their mother and stepfather; and the remaining children (4.9%) in other types of families (e.g., either with their father, father and stepmother, guardian, or grandparents). Parents in our sample were, on average, 35.59 years old (SD = 4.98, ranging from 23 to 60). The questionnaire respondents were primarily mothers (92.2%). Most of the parents had a university degree (63% of mothers and 52.5% of fathers) or had graduated from college or polytechnic school (18.8% of mothers and 26.9% of fathers); 12.1% of mothers and 15.3% of fathers had finished 12 or fewer grades. The teachers were all females of an average age of 45.17 years (SD = 10.70, ranging from 25 to 62). All were educated to be teachers, with three (12.5%) having graduated from teacher’s college, 12 (50.0%) having earned a bachelor’s degree, and nine (37.5%) having completed a master’s degree. Their teaching experience ranged from 0.25 to 41 years (*M* = 21.69, SD = 12.22). Finally, all testers were female professional school psychologists working in the participating schools.

### Measures

The descriptive statistics of raw scores of all study variables (number of valid cases, *M*, SD, min, max, Cronbach’s alpha, and skewness) are presented in Table 1. However, variables concerning relationship quality were standardized (*z*-scores) in all subsequent analyses.

**TABLE 1:** Descriptive statistics.

	*N*	*M*	SD	Cronbach’s alpha	Range		Skewness
					Potential	Actual	
Closeness, parents (T1)	341	4.28	0.45	0.71	1–5	2.88–5	−0.54
Conflict, parents (T1)	341	2.45	0.73	0.80	1–5	1–5	0.60
Closeness, teachers (T1)	342	3.97	0.54	0.73	1–5	2–5	−0.38
Conflict, teachers (T1)	342	1.57	0.70	0.85	1–5	1–4.71	1.62
Closeness, parents (T2)	321	4.22	0.47	0.74	1–5	2.75–5	−0.41
Conflict, parents (T2)	319	2.51	0.75	0.91	1–5	1–5	0.55
Closeness, teachers (T2)	334	3.84	0.60	0.80	1–5	2–5	−0.56
Conflict, teachers (T2)	334	1.64	0.78	0.94	1–5	1–4.71	1.48
Task persistence, teachers (T1)	341	3.60	0.96	0.89	1–5	1–5	−0.58
Task persistence, teachers (T2)	335	3.55	0.97	0.91	1–5	1–5	−0.52
Task persistence, testers (T1)	337	4.14	0.89	0.90	1–5	1–5	−1.18
Task persistence, testers (T2)	336	4.51	0.76	0.91	1–5	1–5	−2.03
Reading (T1)	337	16.56	11.60	0.96	0–75	0–59	0.92
Reading (T2)	341	25.83	12.69	0.97	0–75	0–67	0.60
Spelling (T1)	337	28.64	9.65	0.91	0–40	0–40	−1.51
Spelling (T2)	341	35.35	5.30	0.94	0–40	0–40	−3.61
Addition (T1)	337	5.70	4.23	0.90	0–20	0–20	1.29
Addition (T2)	341	9.90	5.77	0.93	0–23	0–23	0.37
Subtraction (T1)	337	7.15	3.19	0.83	0–20	0–16	−0.07
Subtraction (T2)	341	10.00	3.60	0.86	0–23	0–20	−0.19
Highest education in the family	315	4.61	0.71		1–5	1–5	−1.94
Child gender (1 = girl, 2 = boy)	350	1.47	0.50		1–2	1–2	0.14

#### Parent Questionnaire

##### Parent–Child Relationship (T1 and T2)

To measure parent–child relationship quality (in terms of closeness and conflict), we used the short form of the Child–Parent Relationship Scale (CPRS; Pianta, 1992b). Parents rated 15 items on a five-point Likert-type scale (1 = *Completely disagree*; 5 = *Completely agree*). The questionnaire consisted of two scales: Closeness (8 items; e.g., “*If upset, my child will seek comfort from me*”) and Conflict (7 items; e.g., “*My child and I always seem to be struggling with each other*”).

#### Teacher Questionnaire

##### Teacher–Student Relationship (T1 and T2)

To measure teacher–student relationship quality (in terms of closeness and conflict), we used the short form of the Student–Teacher Relationship Scale (STRS; Pianta, 1992a,^2001^). The Grade 1 teachers rated 15 items on a five-point Likert-type scale (1 = *Completely disagree*; 5 = *Completely agree*). The questionnaire consisted of two scales: Closeness and Conflict. The items were similar to those depicting parent–child relationship quality.

##### Task Persistence (T1 and T2)

Children’s task persistence was measured using the Behavioral Strategy Rating Scale (BSRS; Aunola et al., 2000; Zhang et al., 2011). Teachers rated each student in their classroom. The scale included five items (e.g., “*The child actively tries to manage even the difficult situations or assignments*”) on a five-point Likert-type scale (1 = *Completely disagree;* 5 = *Completely agree*).

#### Tester Questionnaire

##### Task Persistence (T1 and T2)

Children’s task persistence was measured by the BSRS (Aunola et al., 2000; Zhang et al., 2011). Testers rated each student’s task persistence after each individual testing session. The scale included five items that were identical to the items of task persistence and rated by teachers on a five-point Likert-type scale (1 = *Completely disagree;* 5 = *Completely agree*).

#### Child Tests

To measure children’s reading and math skills, we adapted measures from previous studies, such as the First Steps Study (Lerkkanen et al., 2006–2016), the ARMI test battery (Lerkkanen et al., 2006), and the doctoral dissertation of Gedutienė (2008).

##### Reading (T1 and T2)

An individually administered word-reading fluency test based on the Lukilasse test (Häyrinen et al., 1999) and the work of Gedutienė (2008) were used. Each child was presented with a list of 75 real words, divided into three columns. The words ranged from one to four syllables and were written in uppercase letters. The child was instructed to read the words aloud, and the score was based on the number of words read correctly within a 45-s time frame.

##### Spelling (T1 and T2)

An individually administered word-spelling test based on the work of Gedutienė (2008) was used. Each child was presented with a list of 10 real words. The words, which were organized in order of difficulty, ranged from two to three syllables and four to 11 letters in length. The child was asked to spell the words to the best of his or her ability as the tester read the words aloud one at a time. The child had as much time as necessary to write each word. Each word written was scored from 0 to 4 (0 = incorrectly spelled word; 0.5 = one correctly spelled letter, but not the first letter; 1 = only the first letter of the word spelled correctly; 2 = two or more correctly spelled letters; 3 = the word is spelled incorrectly, but contains the correct phonetic structure and/or switched letters; 4 = correctly spelled word). For further analyses, the summative score of the ten scores/words was calculated.

##### Addition (T1 and T2)

The addition test was adapted from Aunola and Räsänen (2007) and modified for the current research purposes. During testing, students were asked to complete as many addition tasks as possible (20 tasks in T1 and 23 tasks in T2) in 60 s for each test. Each student was presented with a stimulus page with the equations and was asked to say the answers aloud. The answers were rated as incorrect or correct (0 = incorrect; 1 = correct); the sum of the correct answers was used as the measure.

##### Subtraction (T1 and T2)

The subtraction test was adapted from Aunola and Räsänen (2007) and modified for the current research purposes. During testing, students were asked to complete as many subtraction tasks as possible (20 tasks at T1 and 23 tasks at T2) in 60 s for each test. Each student was presented with a stimulus page with the equations and was asked to say the answers aloud. Answers were rated as incorrect or correct (0 = incorrect; 1 = correct); the sum of the correct answers was used.

#### Control Variables

**Child gender** was coded as 1 (girl) or 2 (boy).

##### Highest Education Level in the Family

Both parents reported their educational level, and the higher of the two was chosen for further analysis (1 = *have finished 0–8 years*; 2 = *9–10 years*; 3 = *11–12 years*, 4 = *college or polytechnic*, 5 = *university*).

### Analysis Strategy

To answer RQ1 about the latent profiles of parent–child and teacher–student relationship quality (in terms of closeness and conflict), we used latent profile analysis (LPA) separately for T1 and T2. To investigate the stability of the LPA throughout Grade 1 (from T1 to T2), latent transition analysis (LTA) was used. We treated LPA as our preliminary analysis to establish expectations for the LTA analysis. Model estimations were conducted gradually, starting with a one-pattern solution and then increasing the number of patterns step by step to find the best model. Measures were standardized before the analyses. These analyses were conducted using Mplus Version 8.4 (Muthén and Muthén, 1998–2017). Models were estimated using maximum likelihood estimation with robust standard errors (MLR). We used a large number of starting values (STARTS = 10,000 500; STITERATIONS = 500). Because the data were hierarchical in nature (i.e., children nested within classes/teachers), we used the “complex” function of Mplus (Muthén and Muthén, 1998–2017). When applying the TYPE = COMPLEX function, we used “Grade 1 classroom ID” as a clustering variable to compute corrected standard errors and obtain tests of model fit, where the nested structure of the data was taken into account.

The following criteria were used to select the final number of latent profiles: (1) the fit of the model; (2) the number of children assigned to a latent profile/trajectory; and (3) the practical usefulness, theoretical justification, and interpretability of the solution (Muthén, 2003). The fit of the model was evaluated using six indices: (1) the log-likelihood value (Log L), (2) the Akaike information criterion (AIC); (3) the Bayesian information criterion (BIC); (4) the adjusted Bayesian information criterion (ABIC); (5) the Vuong-Lo-Mendell-Rubin likelihood ratio test (VLMRT); and (6) the Lo-Mendell Rubin adjusted likelihood ratio test (ALMRT; Lo et al., 2001). Lower Log L, AIC, BIC, and ABIC values indicated a better model, and significant ALMRT and VLMRT values suggested the need to choose a higher number of groups. In addition, we used two more coefficients—entropy and average posterior probabilities—to help us with the final decision on the number of latent profiles/trajectories. Entropy was used to evaluate overall classification quality; higher entropy values indicate clearer classification and thus are preferred (Muthén, 2003). Average posterior probabilities (PP) were estimated to investigate the precision of the classification of the individual groups/categories, that is, how accurate is the assignment of children to a specific category within overall solution. Values of average PPs higher than 0.70 indicated adequate precision of classification for each individual category (Muthén, 2003).

To answer RQ2, an analysis of variance (ANOVA) in SPSS was conducted to investigate the extent to which latent transitions differed in the means of children’s task persistence and performance. Finally, to answer RQ3 about how profile membership at the beginning of Grade 1 predicts children’s task persistence and performance at the end of Grade 1, we used multiple regression in SPSS. In these regressions, we included autoregressors of the same dependent variable at T1 and added control variables (parental education and child gender) to predict child outcomes (task persistence and performance).

## Results

Psychometric properties of the study variables and a correlation table for all study variables are provided in Tables 1, 2, respectively.

**TABLE 2:** Correlations.

		1	2	3	4	5	6	7	8	9	10	11	12	13	14	15	16	17	18	19	20
1	Closeness, parents (T1)	1																			
2	Conflict, parents (T1)	−0.386**	1																		
3	Closeness, teachers (T1)	0.104	–0.005	1																	
4	Conflict, teachers (T1)	−0.155**	0.133*	−0.420**	1																
5	Closeness, parents (T2)	0.634**	−0.320**	0.064	–0.093	1															
6	Conflict, parents (T2)	−0.342**	0.747**	–0.028	0.133*	−0.382**	1														
7	Closeness, teachers (T2)	0.112*	−0.126*	0.553**	−0.403**	0.171**	–0.096	1													
8	Task persistence, teachers (T1)	0.060	−0.148**	0.390**	−0.528**	–0.024	−0.129*	0.397**	1												
9	Task persistence, teachers (T2)	0.053	−0.177**	0.336**	−0.550**	0.090	−0.138*	0.531**	0.745**	1											
10	Task persistence, testers (T1)	0.073	–0.087	0.246**	−0.269**	0.033	–0.099	0.213**	0.497**	0.414**	1										
11	Task persistence, testers (T2)	0.035	–0.086	0.199**	−0.352**	–0.044	−0.125*	0.237**	0.490**	0.436**	0.591**	1									
12	Reading (T1)	–0.064	–0.024	0.119*	–0.078	–0.073	–0.039	0.084	0.420**	0.385**	0.485**	0.354**	1								
13	Reading (T2)	–0.032	–0.004	0.174**	−0.189**	–0.028	0.032	0.146**	0.552**	0.430**	0.574**	0.468**	0.686**	1							
14	Spelling (T1)	0.004	–0.073	0.120*	–0.090	–0.057	–0.043	–0.011	0.250**	0.287**	0.378**	0.243**	0.358**	0.265**	1						
15	Spelling (T2)	–0.033	–0.056	0.197**	−0.120*	–0.049	–0.049	0.045	0.310**	0.268**	0.418**	0.317**	0.386**	0.401**	0.635**	1					
16	Addition (T1)	−0.118*	–0.012	0.123*	−0.124*	–0.084	–0.002	0.114*	0.421**	0.437**	0.458**	0.408**	0.873**	0.631**	0.376**	0.429**	1				
17	Addition (T2)	–0.021	–0.002	0.296**	−0.286**	–0.008	–0.005	0.236**	0.515**	0.447**	0.551**	0.486**	0.486**	0.722**	0.256**	0.397**	0.562**	1			
18	Subtraction (T1)	0.023	–0.071	0.181**	−0.168**	–0.043	–0.061	0.109*	0.371**	0.400**	0.401**	0.343**	0.385**	0.318**	0.659**	0.596**	0.454**	0.356**	1		
19	Subtraction (T2)	–0.047	–0.097	0.133*	−0.169**	–0.078	−0.111*	0.063	0.372**	0.335**	0.420**	0.407**	0.390**	0.393**	0.639**	0.681**	0.424**	0.433**	0.668**	1	
20	Highest education in the family	0.002	–0.064	0.188**	–0.090	–0.061	–0.077	0.130*	0.201**	0.248**	0.277**	0.244**	0.247**	0.306**	0.194**	0.204**	0.296**	0.370**	0.220**	0.265**	1
21	Child gender (1 = girl, 2 = boy)	–0.098	0.003	−0.231**	0.231**	–0.036	–0.046	−0.256**	−0.215**	−0.216**	–0.053	–0.077	−0.138*	−0.188**	0.191**	0.092	–0.074	−0.144**	0.146**	0.160**	−0.094

**p < 0.01, *p < 0.05.

### RQ1—Latent Profiles in Grade 1 (T1 and T2) and Latent Transitions During Grade 1

Preliminary LPAs for the beginning (T1) and the end (T2) of Grade 1 are presented in Table 3. Based on the Log L, AIC, BIC, and ABIC values, five or six profile solutions were preferred at both time points. The VLMRT and ALMRT indices did not suggest a preference for any solution, whereas entropy values (precision of classification above 0.90) suggested that two-profile solutions were clearly better than the others. We also investigated average PP values. Average PP values above 0.70 would suggest clear classification of cases (Muthén, 2003). Across all solutions at both time points, at least one category in each profile solution had an average PP value below 0.70. The only exception was a two-profile solution at both time points with average PP values being above 0.70 at both measurement points. Most importantly, we had a preliminary look at the plots of all profile solutions, and it appeared that the two-profile solution exhibited the clearest pattern at both time points. Adding more groups caused the groups to differ by close to average mean levels (i.e., not distinct patterns), thus, made them difficult to interpret. In addition, increasing the number of groups made some of the groups relatively small, thus, difficult to justify theoretically and conduct further analyses. The latter three reasons (entropy and average PPs, interpretability, and the number of children in each profile category) led us to the decision to choose a two-profile solution at both time points.

**TABLE 3:** Fit indices for latent profile analysis for 1–6 latent profiles at the beginning (T1) and end (T2) of Grade 1.

Criterion	One profile	Two profiles	Three profiles	Four profiles	Five profiles	Six profiles
**Beginning of Grade 1**						
Log-likelihood	−1,935.4	−1,855.5	−1,834.4	−1,795.8	−1,764.0	−1,768.2
Parameters	8	13	18	23	28	33
AIC	3,886.86	3,736.94	3,704.85	3,637.52	3,584.05	3,602.44
BIC	3,917.73	3,787.09	3,774.3	3,726.25	3,692.07	3,729.75
ABIC	3,892.35	3,745.85	3,717.19	3,653.29	3,603.24	3,625.06
Entropy		0.947	0.756	0.881	0.897	0.808
VLMRT (*p*-value)		0.184	0.394	0.450	0.716	0.646
ALMRT (*p*-value)		0.192	0.403	0.455	0.719	0.649
Profile 1 (%)		92	73	62	2	7
Profile 2 (%)		8	7	7	59	49
Profile 3 (%)			18	4	4	<1
Profile 4 (%)				27	7	9
Profile 5 (%)					28	19
Profile 6 (%)						15
***End of Grade 1***						
Log-likelihood	−1,856.0	−1,767.9	−1,748.7	−1,716.8	−1,660.6	−1,652.4
Parameters	8	13	18	23	28	33
AIC	3,727.94	3,561.71	3,533.46	3,479.57	3,377.10	3,370.76
BIC	3,758.53	3,611.41	3,602.28	3,567.50	3,484.15	3,496.92
ABIC	3,722.15	3,570.17	3,545.18	3,494.54	3,395.33	3,392.24
Entropy		0.918	0.696	0.792	0.846	0.813
VLMRT (*p*-value)		0.371	0.518	0.688	0.707	0.675
ALMRT (*p*-value)		0.381	0.524	0.691	0.709	0.675
Profile 1 (%)		88	19	49	10	25
Profile 2 (%)		12	12	31	49	4
Profile 3 (%)			69	12	11	44
Profile 4 (%)				8	4	10
Profile 5 (%)					27	12
Profile 6 (%)						4

*AIC, Akaike information criterion; BIC, Bayesian information criterion; ABIC, adjusted Bayesian information criterion; VLMRT, Vuong-Lo-Mendell-Rubin likelihood ratio test; ALMRT, Lo-Mendell-Rubin adjusted likelihood ratio test.*

**FIGURE 1:**
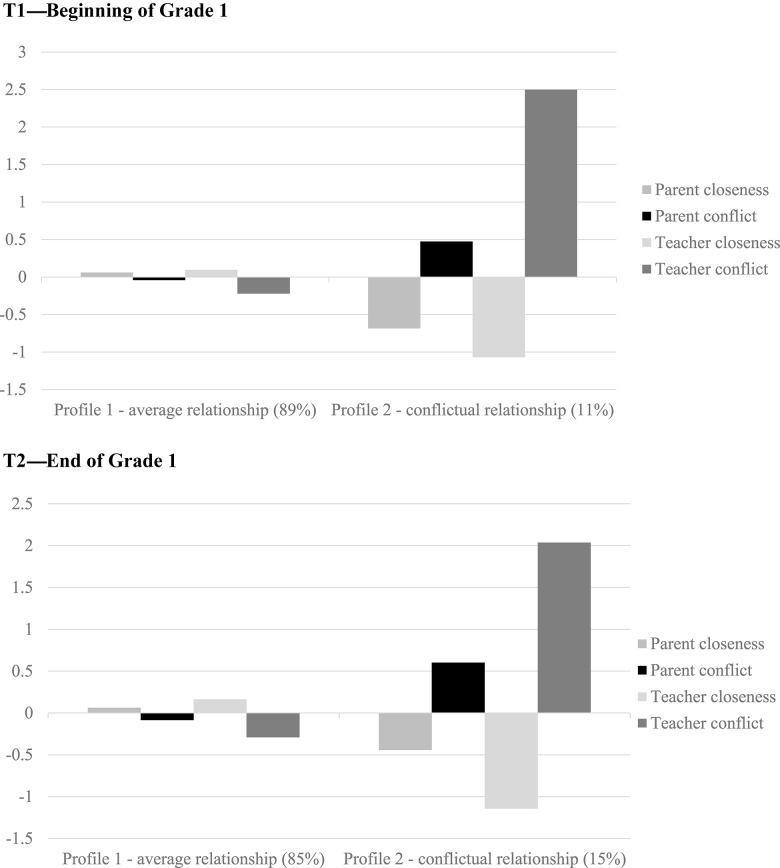
Standardized means of latent profiles at the beginning (T1) and end (T2) of Grade 1.

Based on these LPA results, LTA was conducted. The results of the LTA (Table 4) further supported a two-profile solution across time points (based on entropy, proportion, and meaningfulness of latent groups). The profiles are presented in Figure 1. The larger profile at each time point (92% at T1 and 88% at T2) can be characterized by the average level (less than a 0.5 standard deviation) of any of the dimensions constituting the profile (parent and teacher closeness and conflict); thus, it was labeled the *average relationship* profile. The other profile had lower-than-average closeness with both parents (close to half a standard deviation at T1 and T2) and teachers (more than one standard deviation below average at T1 and T2) and higher-than-average conflict with parents (around half a standard deviation at T1 and T2) and teachers (more than two standard deviations above average). The latter profile was labeled the *conflictual relationship* profile (8% at T1 and 12% at T2).

**TABLE 4:** Fit indices for latent transition analysis for 2–5 latent profiles at the end of Grade 1.

Criterion	Two profiles	Three profiles	Four profiles	Five profiles
Log-likelihood	−3,573.5	−3,472.7	−3,367.4	−3,289.9
Parameters	27	40	55	72
AIC	7,200.93	7,025.50	6,844.83	6,723.76
BIC	7,305.09	7,179.81	7,057.01	7,001.53
ABIC	7,219.44	7,052.92	6,882.53	6,773.12
Entropy	0.948	0.885	0.919	0.897
***Beginning of Grade 1 (T1)***				
Profile 1 (%)	89	63	59	14
Profile 2 (%)	11	10	4	30
Profile 3 (%)		27	29	47
Profile 4 (%)			7	7
Profile 5 (%)				2
***End of Grade 2 (T2)***				
Profile 1 (%)	85	61	4	15
Profile 2 (%)	15	12	58	10
Profile 3 (%)		35	10	4
Profile 4 (%)			27	44
Profile 5 (%)				27

*AIC, Akaike information criterion; BIC, Bayesian information criterion; ABIC, adjusted Bayesian information criterion.*

**FIGURE 2:**
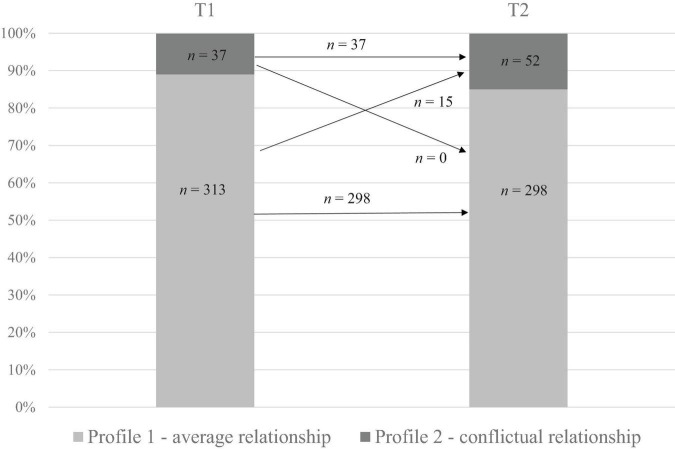
Latent transition probabilities between beginning (T1) and end (T2) of Grade 1.

Also with respect to RQ1, three latent transitions were identified. The results of LTA indicated that most children stayed in similar profiles across both time points (see Table 5 and Figure 2). In particular, all the children in the *conflictual relationship* profile at T1 remained in this profile at T2, and many children in the *average relationship* profile stayed in this profile across T1 and T2 (with a 95% chance of remaining in the same pattern). Most interestingly, none of the children moved from the *conflictual relationship* to the *average relationship* profile, whereas 15 children moved from the *average relationship* to the *conflictual relationship* profile over time. Consequently, we obtained three trajectories during the Grade 1 school year: *average relationship trajectory* (*n* = 298; 85.14%), *conflictual relationship trajectory* (*n* = 37; 10.57%), and *declining relationship trajectory* (*n* = 15; 4.29%).

**TABLE 5:** Latent transition probabilities between the beginning (T1) and end (T2) of Grade 1.

Beginning of Grade 1 (T1)	End of Grade 1 (T2)	
	Profile 1 Average relationship (85%)	Profile 2 Conflictual relationship (15%)
Profile 1 Average relationship (89%)	0.948	0.052
Profile 2 Conflictual relationship (11%)	0.000	1.000

As additional analyses, we ran a set of one-way ANOVAs (Table 6) to explore how transitions would differ for parents versus teachers in terms of the separate dimensions of closeness and conflict. The results showed that at the beginning of Grade 1 (T1), children following the *average relationship trajectory* were rated higher in closeness and lower in conflict by both parents and teachers than children on the *conflictual relationship trajectory*. Also, children following the *conflictual relationship trajectory* were rated as having higher conflict and lower closeness by teachers than children on the *declining relationship trajectory*, although they did not differ in closeness and conflict when rated by parents. At T2, however, closeness with parents did not differ among the three trajectories. Conflict with parents was higher for the *conflictual relationship trajectory* when compared to the *average relationship trajectory*. Similarly to T1, the *average relationship trajectory* was higher in closeness with teachers and lower in conflict with teachers in comparison to the *conflictual relationship trajectory.* Most interestingly, in contrast to the T2 results for teacher–student closeness and conflict, the *declining relationship trajectory* was lower in closeness and higher in conflict than the *average relationship trajectory* (and no longer different from the *conflictual relationship trajectory*), indicating that changes in relationship quality with teachers (versus parents) might be responsible for the changes in/worsening of the overall patterns of relationships.

**TABLE 6:** Mean differences in latent transitions regarding children’s closeness and conflict with parents and teachers.

	Transition 1		Transition 2		Transition 3			
	Average relationship trajectory (*n* = 298; 85.14%)		Conflictual relationship trajectory (*n* = 37; 10.57%)		Declining relationship trajectory (*n* = 15; 4.29%)			
	*M*	SD	*M*	SD	*M*	SD	*F*	*p*
Closeness, parents (T1)	0.08^a^	0.96	−0.52^a^	1.08	−0.32	1.18	6.521	**0.002**
Conflict, parents (T1)	−0.09^a^	0.99	0.56^a^	0.99	0.48	0.81	8.678	**<0.001**
Closeness, teachers (T1)	0.07^a^	0.93	−0.76^ab^	1.16	0.47^b^	1.13	13.815	**<0.001**
Conflict, teachers (T1)	−0.28^a^	0.61	2.25^ab^	0.86	0.08^b^	0.48	256.346	**<0.001**
Closeness, parents (T2)	0.05	0.99	−0.22	1.11	−0.39	0.87	2.219	0.110
Conflict, parents (T2)	−0.08^a^	0.97	0.48^a^	0.99	0.39	1.34	5.993	**0.003**
Closeness, teachers (T2)	0.17^ab^	0.91	−0.94^a^	0.97	−0.99^b^	0.99	31.519	**<0.001**
Conflict, teachers (T2)	−0.34^ab^	0.56	1.85^a^	0.81	2.03^b^	0.85	295.087	**<0.001**

*Mean scores that share the same superscript are statistically significantly different. Bonferroni was used for all other indicators. Bold font indicates significant results at the p < 0.05 level.*

### RQ2—The Role of Learning Variables in Latent Transitions

To answer RQ2, we compared mean level differences between trajectories in terms of children’s performance and task persistence. One-way ANOVA results indicated (Table 7) that teacher-rated task persistence at T1 was the lowest in the *conflictual relationship trajectory* in comparison to both the *average relationship trajectory* and *declining relationship trajectory.* In contrast, task persistence at T2 was the highest in the *average relationship trajectory* and significantly differed from the other two. As for tester-rated task persistence, at T1 and T2, only the *average relationship trajectory* scored higher than the *conflictual relationship trajectory.*

**TABLE 7:** Mean differences in latent transitions regarding children’s task persistence and performance.

	Transition 1		Transition 2		Transition 3			
	Average relationship trajectory (*n* = 298; 85.14%)		Conflictual relationship trajectory (*n* = 37; 10.57%)		Declining relationship trajectory (*n* = 15; 4.29%)			
	*M*	SD	*M*	SD	*M*	SD	*F*	*p*
Task persistence, teacher rated (T1)	3.74^a^	0.89	2.55^ab^	0.85	3.47^b^	0.82	28.806	**<0.001**
Task persistence, teacher rated (T2)	3.75^ab^	0.86	2.34^a^	0.77	2.53^b^	0.69	54.900	**<0.001**
Task persistence, tester rated (T1)	4.22^a^	0.83	3.67^a^	1.15	3.88	1.09	6.623	**0.002**
Task persistence, tester rated (T2)	4.58^a^	0.69	3.98^a^	1.08	4.40	0.82	10.424	**<0.001**
Reading (T1)	16.76	11.45	16.03	13.50	14.07	10.34	0.422	0.656
Reading (T2)	26.05	12.46	24.31	15.40	24.47	10.89	0.375	0.687
Spelling (T1)	29.05	9.30	25.80	12.39	27.57	8.47	1.870	0.156
Spelling (T2)	35.65^a^	4.86	32.60^a^	8.46	35.60	1.80	5.280	**0.006**
Addition (T1)	5.79	4.15	4.83	4.85	6.13	4.42	0.884	0.414
Addition (T2)	10.06	5.74	8.37	5.85	10.73	5.79	1.502	0.224
Subtraction (T1)	7.17^a^	3.11	6.17^b^	3.67	9.20^ab^	2.68	4.845	**0.008**
Subtraction (T2)	10.10	3.50	8.77	4.34	11.20	3.19	3.011	0.051
Highest education in the family	4.64	0.66	4.37	1.01	4.46	0.74	2.146	0.119
Child gender (1 = girl, 2 = boy)	1.41^ab^	0.49	1.76^a^	0.43	1.80^b^	0.41	12.028	**<0.001**

*Mean scores that share the same superscript are statistically significantly different. Bonferroni was used for all other indicators. Bold font indicates significant results at the p < 0.05 level.*

Finally, overall, the children’s performance in reading, spelling, addition, and subtraction did not differ according to relationship trajectory. The only two exceptions were (1) spelling at T2, where the *average relationship trajectory* scored higher than the *conflictual relationship trajectory*, and (2) subtraction at T1, where the *declining relationship trajectory* scored the highest and differed from the other two trajectories.

The additional analyses indicated that parental education did not differ across trajectories (see Table 7), whereas child gender did [χ^2^(2, *N* = 350) = 22.692, *p* < 0.05]. In particular, we ran a chi-square test and found that there were more girls than boys on the *average relationship trajectory* (175 girls, 123 boys) than on the *conflictual relationship trajectory* (9 girls, 28 boys) or the *declining relationship trajectory* (3 girls, 12 boys).

Given a strong relation between gender and trajectories, and unexpected relations between trajectories and performance in spelling and subtraction, we conducted further analyses to test gender differences in children’s performance. As presented in Table 8, we found that, overall, girls performed better than boys in reading and spelling, as significant differences favoring girls were found in reading at T1 and spelling at T1 and T2. In contrast, boys performed better than girls in addition and subtraction, scoring significantly higher in addition at T1 and T2, and in subtraction at T2. Thus, gender differences in performance may partly explain differences in performance between the trajectories.

**TABLE 8:** Gender differences in children’s performance.

	Girls		Boys				
	(*n* = 187)		(*n* = 163)				
	*M*	SD	*M*	SD	*t*	*df*	*p*
Reading (T1)	18.055	11.046	14.847	12.026	2.552	335	**0.011**
Reading (T2)	26.685	12.499	24.805	12.904	1.358	335	0.175
Spelling (T1)	30.330	8.340	26.707	10.668	3.494	335	**0.001**
Spelling (T2)	36.056	4.063	34.525	6.367	2.659	335	**0.008**
Addition (T1)	4.950	3.569	6.573	4.757	−3.569	335	**<0.001**
Addition (T2)	9.117	5.201	10.805	6.225	−2.709	335	**0.007**
Subtraction (T1)	6.883	2.771	7.471	3.602	−1.690	335	0.092
Subtraction (T2)	9.466	3.106	10.622	4.013	−2.974	335	**0.003**

*Bold font indicates significant results at the p < 0.05 level.*

### RQ3—Predicting Children’s Learning at the End of Grade 1 (T2)

To answer RQ3, we predicted children’s learning outcomes at T2 by means of their profile membership at T1 after controlling for the autoregressor (the same child outcome measured at T1), parent education, and child gender. The results show (Table 9) that profile membership significantly predicted task persistence rated by the teacher, by the tester, and children’s spelling and subtraction skills. In particular, children with an *average relationship* profile were more likely to develop better task persistence, spelling, and subtraction skills during Grade 1 compared to children with a *conflictual relationship* profile. The development of reading and addition skills during Grade 1 was the same for both profiles.

**TABLE 9:** Multiple regressions predicting children’s task persistence and performance.

Variable	*B*	SE	β	*t*	*p*
**Task persistence, teacher rated (T2)** (*R*^2^ = 0.573)					
Autoregressor	0.661	0.044	0.639	15.141	**< 0.001**
Profiles at T1 (1 = *average relationship*, 2 = *conflictual relationship*)	−0.491	0.140	−0.145	−3.513	**0.001**
Highest education in the family	0.165	0.054	0.120	3.053	**0.002**
Child gender (1 = girl, 2 = boy)	−0.093	0.076	−0.048	−1.226	0.221
**Task persistence, tester rated (T2)** (*R*^2^ = 0.364)					
Autoregressor	0.466	0.044	0.535	10.678	**<0.001**
Profiles at T1 (1 = *average relationship*, 2 = *conflictual relationship*)	−0.328	0.133	−0.119	−2.468	**0.014**
Highest education in the family	0.090	0.054	0.082	1.649	0.100
Child gender (1 = girl, 2 = boy)	−0.056	0.075	−0.036	−0.751	0.453
**Reading (T2)** (*R*^2^ = 0.768)					
Autoregressor	0.914	0.032	0.841	28.578	**<0.001**
Profiles at T1 (1 = *average relationship*, 2 = *conflictual relationship*)	−1.616	1.311	−0.036	−1.233	0.218
Highest education in the family	1.964	0.530	0.109	3.707	**<0.001**
Child gender (1 = girl, 2 = boy)	1.145	0.745	0.045	1.536	0.126
**Spelling (T2)** (*R*^2^ = 0.567)					
Autoregressor	0.371	0.023	0.651	15.931	**<0.001**
Profiles at T1 (1 = *average relationship*, 2 = *conflictual relationship*)	−2.237	0.779	−0.133	−2.874	**0.004**
Highest education in the family	1.480	0.317	0.189	4.670	**<0.001**
Child gender (1 = girl, 2 = boy)	0.041	0.443	0.004	0.093	0.926
**Addition (T2)** (*R*^2^ = 0.463)					
Autoregressor	0.882	0.063	0.634	14.074	**<0.001**
Profiles at T1 (1 = *average relationship*, 2 = *conflictual relationship*)	−0.876	0.912	−0.042	−0.960	0.338
Highest education in the family	0.984	0.363	0.120	2.711	**0.007**
Child gender (1 = girl, 2 = boy)	0.328	0.525	0.028	0.626	0.532
**Subtraction (T2)** (*R*^2^ = 0.525)					
Autoregressor	0.737	0.049	0.641	15.133	**<0.001**
Profiles at T1 (1 = *average relationship*, 2 = *conflictual relationship*)	−1.362	0.546	−0.104	−2.494	**0.013**
Highest education in the family	0.714	0.218	0.138	3.277	**0.001**
Child gender (1 = girl, 2 = boy)	0.872	0.310	0.118	2.816	**0.005**

*Bold font indicates significant results at the p < 0.05 level.*

## Discussion

The present study applied a person-oriented approach to longitudinal multiple-respondent data to investigate profiles and trajectories of parent–child (rated by parents) and teacher–student (rated by teachers) relationships during Grade 1. Two latent profiles were found at both the beginning and end of Grade 1: (1) *average relationship* with parents and teachers (89% at T1 and 85% at T2) and (2) *conflictual relationship* with parents and teachers (11% at T1 and 15% at T2). These profiles were highly stable throughout Grade 1, except for 15 children moving from the *average relationship* profile to the *conflictual relationship* profile. This *declining trajectory* can be characterized by low teacher-reported relationship quality and low task persistence at the end of Grade 1, whereas the performance of children on this trajectory was no worse than that of the rest of the children. Finally, children with a conflictual relationship with their parents and teachers at the beginning of Grade 1 performed worse on the spelling and subtraction tasks and demonstrated lower task-persistent behavior at the end of Grade 1 than those with average (good) relationships with parents and teachers.

### Profiles of the Relationship Quality With Parents and Teachers and Their Transitions During Grade 1

The results show that the majority of Grade 1 children are in good relationships with their interpersonal environments. That is, for 85–89% of the children, parents, and teachers reported good relationships, whereas only for 11–15% of children did parents and teachers report conflictual relationships. Our results are in accordance with the findings of many previous studies that most children have close and non-conflictual relationships with their parents and teachers (Spilt et al., 2012; Miller-Lewis et al., 2014; Heatly and Votruba-Drzal, 2017; Valiente et al., 2019). Although we called our profiles and transitions “average” due to their average values in the standardized solution, in fact, the overall means of raw scores across Grade 1 for parent- and teacher-perceived closeness was between 3.84 and 4.28 (maximum 5) and for parent- and teacher-perceived conflict was between 1.57 and 2.51 (maximum 5). This suggests that for most children, their relationships with parents and teachers can be categorized as close and not conflictual, and only up to around 15% of children deviate from the average enough to form a distinctly separate group. These results align with our expectations of finding one adaptive (average) profile; the results also clarified our expectations concerning finding any non-adaptive profiles. We did not find separate profiles with extreme values for closeness and conflict exclusively for parents or teachers; instead, closeness and conflict deviated from the average for both parents and teachers. However, it is worth emphasizing that it was mostly the reports of teachers (versus parents) that were responsible for the distinct *conflictual relationship* profile. One explanation for these results might be that parents, overall, tend to report close and not conflictual relationships with their Grade 1 students because they may not have much of a frame of reference for comparison of their relationships with their children. As for teachers, who are dealing with the whole class, it is easier to distinguish differences in relationship quality among various students.

The profiles were highly stable during the Grade 1 school year, except for 15 children who moved from the *average relationship* to the *conflictual relationship* profile. The three trajectories [*average relationship trajectory* (85.14%), *conflictual relationship trajectory* (10.57%), and *declining relationship trajectory* (4.29%)] are in line with many previous variable-oriented studies showing that closeness and conflict with parents and teachers tend to be stable throughout primary school and beyond (Pianta, 1999; Heatly and Votruba-Drzal, 2019). As for person-oriented research, our results are somewhat similar to those of Miller-Lewis et al. (2014), who found two trajectories of teacher–student relationships from preschool to Grade 2. In particular, the authors used a composite score of relationship quality with teachers (in terms of closeness and reversed conflict) and found that the majority of children were in stable, high-quality relationships with teachers, whereas for some children, their relationship with their teachers was of moderate quality and declining. The present study complements this result by reporting similar trajectories when relationship quality with parents is considered. In contrast, our results somehow contradict those obtained by certain other researchers (O’Connor et al., 2011; Spilt et al., 2012; Valiente et al., 2019), who found more than one non-stable trajectory. This

is understandable, however, as these studies followed children across longer periods of time. Although our study has specifically focused on the trajectories at the very start of children’s school career (Grade 1), following parent and teacher reports for a longer period of time remains a task for future research.

To complement our findings identifying three trajectories, our additional analyses explored how these three trajectories would differ on the basis of separate components/dimensions of relationship quality (in terms of closeness and conflict of parents and teachers). Overall, the *declining relationship trajectory* did not differ from the other two based on parent reports of closeness and conflict at both time points, but the situation was different concerning teacher reports. In particular, at the start of Grade 1, the *declining relationship trajectory* scored significantly higher in closeness and significantly lower in conflict. However, the situation changed at the end of Grade 1, when teacher reports for the *declining relationship trajectory* were the lowest for closeness and the highest for conflict (thus becoming more similar to those for the *conflictual relationship trajectory* and significantly different from those for the *average relationship trajectory*). These results emphasize the importance of children’s relationship with teachers regarding the overall relationship quality with interpersonal environments starting in Grade 1. This result is also not surprising because at the start of Grade 1, teachers meet their students for the first time and might not have had the time/opportunity to get to know them and, thus, base their judgments on their first impressions of the child; also, students might be in the process of adapting to their new environments and not yet have set patterns of relationships with their teachers. By the end of Grade 1, however, teachers have had more time to work with students in their classes and, thus, the relationship quality and judgment of it could change due to, for example, children’s response to instruction, their behavior, etc., during the year.

### Differences in Task Persistence and Performance Between Trajectories of Relationship Quality

To answer RQ2, we investigated how mean differences in task persistence and performance would differ across trajectories. Among these learning-related factors of children, the role of task persistence was the clearest in differentiating among the three trajectories. Task persistence was rated by two reporters: Grade 1 teachers and school psychologists who tested the children. Both teachers and testers consistently rated children on the *average relationship trajectory* as significantly more task persistent than those on the *conflictual relationship trajectory*. Interestingly, differences between teachers’ and testers’ ratings were found with respect to the *declining relationship trajectory*. In particular, testers’ ratings of task persistence for children on the *declining relationship trajectory* did not differ from other trajectories. As for teacher’s ratings, at the beginning of Grade 1, the *declining trajectory* was more similar to the *average relationship trajectory* and had a significantly higher task persistence than the *conflictual relationship trajectory*. At the end of Grade 1, teachers rated children on the *declining trajectory* as more similar to children on the *conflictual trajectory* and significantly lower than children on the *average relationship trajectory*. Overall, these results suggest that the *declining trajectory* can be characterized by a lowering of teachers’ perceptions of children’s task persistence in school tasks over the course of Grade 1. Similar results were obtained in previous studies (Heatly and Votruba-Drzal, 2019), including a meta-analysis by Roorda et al. (2011), suggesting that associations of teacher–relationship quality with engagement (a concept somewhat close to our task persistence) range from medium to large. Thus, our study seems to confirm that teachers pay special attention to children’s learning behavior when evaluating the quality of their relationships with their students.

Another learning-related factor is children’s academic performance, with reading, spelling, addition, and subtraction being the essential skills taught in Grade 1. Overall, our results showed that children’s skills (reading, spelling, addition, and subtraction) did not differentiate the trajectories as clearly as task persistence. However, two notable exceptions were found. First, spelling at the end of Grade 1 was significantly higher for the *average relationship* than the *conflictual relationship trajectory*, with the *declining relationship trajectory* falling in-between without significantly differing from either. Second, and most surprisingly, subtraction skills at the beginning of Grade 1 for the *declining trajectory* were significantly higher than for the other two trajectories. Taken together, contrary to our expectations, these results suggest that children on the *declining relationship trajectory* are no lower in their performance than the rest of the children and can even possess higher skills than the rest. These results are somewhat aligned with those of the meta-analysis by Roorda et al. (2011), who reported small to medium associations between teacher–student relationships and performance. Therefore, academic performance as such may not necessarily be the defining factor of the quality of children’s relationships with their parents and teachers in Grade 1.

Finally, in our additional analyses, socio-demographic control factors revealed some interesting results. Following a certain trajectory did not differ according to the highest education level in the family, whereas child gender differentiated the trajectories. In particular, in accordance with previous research (Roorda et al., 2011), there were significantly more boys on the *conflictual relationship* and *declining relationship trajectories* than on the *average relationship trajectory*, suggesting that boys are at greater risk of developing conflictual relationships with their interpersonal environments. Thus, more attention needs to be paid to the reactions that boys evoke from their parents and teachers in order to diminish the chances that boys will develop conflictual relationships with their interpersonal environments. Our additional analyses also revealed that girls scored higher in reading and spelling skills than boys, and boys scored higher in addition and subtraction than girls. This result further supports our findings that performance alone is not a defining factor in the differentiation between the trajectories of relationships with parents and teachers, and child gender—being boy, in particular—is a risk factor for conflictual relationship with parents and teachers.

Taken together, the results seem to confirm that certain child characteristics are more likely to evoke certain responses from their interpersonal environments (Scarr and McCartney, 1983; Sameroff, 2010; Nurmi, 2012). We found support for children’s behavior being more consistently related to the quality of their relationships with parents and teachers than to academic performance (Roorda et al., 2011; Silinskas et al., 2015). Interestingly, child gender also emerged as one of the strongest predictors, suggesting that boys are at greater risk of conflictual relationships with their interpersonal environments.

### Profiles of Relationship Quality as Predictors of Children’s Learning Outcomes

RQ3 asked how profiles of the quality of relationships with parents and teachers at the start of Grade 1 predict children’s task persistence and performance at the end of Grade 1. We found that profiles of relationship quality consistently predicted both teacher and tester ratings of children’s task persistence. In particular, children belonging to the *conflictual relationship* profile had lower task persistence at the end of Grade 1 in comparison to the *average relationship* profile. These results are in accordance with many previous studies (Estrada et al., 1987; Pianta et al., 1991; Gregory and Rimm-Kaufman, 2008) and meta-analysis (Roorda et al., 2011). Theoretically, the results confirm that both parents and teachers are key agents in children’s academic development (Bronfenbrenner, 1979; Bronfenbrenner and Morris, 2006) and that, besides general instructional support, close and non-conflictual relationships may satisfy children’s needs for belonging and competence (Ryan and Deci, 2000, 2020), thus strengthening their task-persistent behavior in learning situations.

Concerning children’s performance, we found that their reading and other skills were not dependent on their relationship profiles with parents and teachers at the start of Grade 1, confirming that relationship quality did not relate to the development of the children’s basic academic development. In contrast, we found that children with the *average relationship profile* at the start of Grade 1 developed better spelling and subtraction skills than those with the *conflictual relationship profile*, suggesting the need for differentiation between basic and more difficult/advanced academic skills. Our results somewhat contradict previous studies that look at the distinction between reading-related and math-related skills (Ly et al., 2012; McCormick et al., 2013; Hajovsky et al., 2017; Zee et al., 2021). However, previous research has shown that spelling skills develop more slowly than reading skills (Leppänen et al., 2006; Landerl and Wimmer, 2008; Georgiou et al., 2020). The same distinction fits addition versus subtraction skills. Thus, good relationships with parents and teachers may promote more advanced skills, such as spelling (versus reading) and subtraction (versus addition). This distinction between basic and more advanced skills may partly clarify the suggestion of previous literature (Roorda et al., 2011) that associations between relationship quality and performance are somewhat more modest (small to medium) compared to those with engagement (medium to large). However, further comparisons of the effects on reading versus math and/or basic versus advanced skills is a task for future research.

## Limitations and Future Directions

There are some limitations that should be considered. First, even though we used longitudinal data, our results should be interpreted carefully, as only experimental studies can determine the direction of effects. Second, the teacher–student relationship and students’ task persistence were rated by their teachers, which may have exposed our results to common-method bias (Roorda et al., 2011). However, for task persistence, we have also used tester reports about children’s behavior during testing situations. These reports showed similar tendencies as did teacher reports. Third, the present study focused on children’s relationship quality during Grade 1. Future studies should investigate whether the same patterns can be identified in older children (e.g., further along in primary school). In following patterns of relationships over a longer period, more and different changes might occur. Finally, in interpreting and generalizing the findings, the specific cultural context of Lithuania should be taken into account—specifically, that children enter Grade 1 in the year of their seventh birthday, and then the systematic learning of reading, spelling, and math starts. Children get a new Grade 1 teacher, and new bonds and relationships with the teacher form.

## Conclusion and Practical Implications

The present study considered not only the quality of child–teacher and child–parent relationships but also both interpersonal environments simultaneously (Bronfenbrenner, 1979; Bronfenbrenner and Morris, 2006), gathered data from multiple informants, and considered non-linear associations by applying a person-oriented approach (Bergman et al., 2003). Consequently, the current study draws a larger picture of the interplay between relationship quality with parents and teachers, its development during Grade 1, and associations with children’s task persistence and performance. Based on our results, it is important to acknowledge that the characteristics that children display at the beginning of Grade 1 can shape their relationships with their important adults (parents and teachers) and that the relationships formed during the transition to and over the course of Grade 1 might be very important for the development of children’s task persistence and performance of more advanced skills (e.g., spelling and subtraction). Thus, parents and teachers of Grade 1 students should become aware of how children’s learning-related characteristics might affect the quality of their relationship with their children. Moreover, relationship quality in terms of closeness and conflict should be taken seriously because it relates to their task persistence and performance at the end of Grade 1.

## Data Availability Statement

The raw data supporting the conclusions of this article are not publicly available, but are available from the corresponding author, upon reasonable request.

## Ethics Statement

The studies involving human participants were reviewed and approved by the Human Sciences Ethics Committee, University of Jyvaskyla. Written informed consent to participate in this study was provided by the participants’ legal guardian/next of kin.

## Author Contributions

GS conducted the analyses, wrote the first draft, and finalized the manuscript. EK contributed to writing and commented throughout the process of preparation of the manuscript. Both authors contributed to the article and approved the submitted version.

## Conflict of Interest

The authors declare that the research was conducted in the absence of any commercial or financial relationships that could be construed as a potential conflict of interest.

## Publisher’s Note

All claims expressed in this article are solely those of the authors and do not necessarily represent those of their affiliated organizations, or those of the publisher, the editors and the reviewers. Any product that may be evaluated in this article, or claim that may be made by its manufacturer, is not guaranteed or endorsed by the publisher.
